# Quantum Transport of the 2D Surface State in a Nonsymmorphic Semimetal

**DOI:** 10.1021/acs.nanolett.0c04946

**Published:** 2021-04-09

**Authors:** Xue Liu, Chunlei Yue, Sergey V. Erohin, Yanglin Zhu, Abin Joshy, Jinyu Liu, Ana M Sanchez, David Graf, Pavel B. Sorokin, Zhiqiang Mao, Jin Hu, Jiang Wei

**Affiliations:** † Institutes of Physical Science and Information Technology, Anhui University, Hefei 230601, China; ‡ Department of Physics and Engineering Physics, Tulane University, New Orleans, Louisiana 70118, United States; § National University of Science and Technology “MISIS”, Leninsky Prospect 4, Moscow 119049, Russian Federation; ∥ Moscow Institute of Physics and Technology (State University), 9 Institutskiy per., Dolgoprudny, Moscow Region 141701, Russian Federation; ⊥ Department of Physics, University of Warwick, Coventry CV4 7AL, United Kingdom; # National High Magnetic Field Lab, Tallahassee, Florida 32310, United States; ○ Department of Physics, Pennsylvania State University, University Park, Pennsylvania 16802, United States; △ Department of Physics, Institute for Nanoscience and Engineering, University of Arkansas, Fayetteville, Arkansas 72701, United States

**Keywords:** 2D topological nodal line semimetal, nonsymmorphic symmetry, surface transport, SdH quantum oscillation

## Abstract

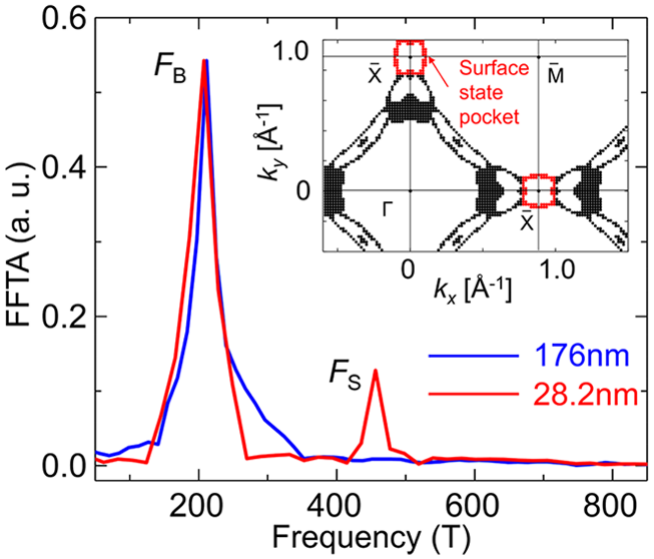

In a topological semimetal with Dirac or Weyl points, the bulk-boundary correspondence principle predicts a gapless edge mode if the essential symmetry is still preserved at the surface. The detection of such topological surface state has been considered as the fingerprint prove for crystals with nontrivial topological bulk band. On the contrary, it has been proposed that even with symmetry broken at the surface, a new surface band can emerge in nonsymmorphic topological semimetals. The symmetry reduction at the surface lifts the bulk band degeneracies and produces an unusual “floating” surface band with trivial topology. Here, we first report quantum transport probing to ZrSiSe thin flakes and directly reveal transport signatures of this new surface state. Remarkably, though topologically trivial, such a surface band exhibits substantial two-dimensional Shubnikov–de Haas quantum oscillations with high mobility, which signifies a new protection mechanism and may open applications for quantum computing and spintronic devices.

The surface electronic state has been a central focus of condensed matter physics. Distinct electrical properties from a well-protected surface (or edge) state, such as quantum Hall or quantum spin Hall effects, provide ample opportunities for the surface state-based device applications.^1−5^ The conventional surface states, resulting from the termination of the three-dimensional (3D) bulk periodic potential, are susceptible to defects or impurities, which appear nearly inevitably in crystals. Recently, there have been significant breakthroughs in the search for robust surface states along with the search for new topological quantum materials. Topological surface states found in bulk topological insulators^6−11^ eliminates backscattering due to the spin-momentum locking, which is originated from the chiral linear energy dispersion as protected by the time-reversal or lattice symmetries. In Weyl semimetals and Dirac semimetals,^1,2^ unusual surface states appear as disconnected or jointed Fermi arcs curving in opposite directions, respectively. Experimentally, there has been extensive characterization on the transport properties of surface states in topological insulators.^7^ For 3D topological semimetals, Weyl orbit on the surface of bulk Dirac semimetal Cd_3_As_2_ has recently been observed showing quantum oscillations^12,13^ and quantum Hall effect^14,15^ in the nanostructured device owing to the enhanced transport signal ratio of surface to bulk. In contrast to the above topologically protected surface states, a new 2D floating surface state can emerge in ZrSi*M* (*M* = S, Se, or Te) nonsymmorphic topological semimetals.^4^ Such a new surface state originates from the symmetry reduction at the surface, thus distinct from the well-known “conventional” topological surface state arising from the bulk-boundary correspondence principle in topological materials.

ZrSi*M* belongs to the recently discovered *WHM*-type (*W* = Zr, Hf, or rare-earth; *H* = Si, Ge, Sn)^16−25^ topological semimetal family. These materials crystallize in layered tetragonal structure (Figure 1a) and possess two types of Dirac states: the nodal-line Dirac state protected by the *C*_2*v*_ symmetry and gapped by spin–orbit coupling^16,17^ and the 2D gapless nodal-point Dirac state protected by the nonsymmorphic symmetry.^16,26−28^ The different combinations of *W*, *H*, and *M* elements further give rise to high tunability in spin–orbit coupling,^29−31^ magnetism,^22,23^ and structural dimensionality,^17,21,30^ leading to rich electronic properties of various *WHM*s such as large magnetoresistance,^32,33^ high Dirac Fermion density,^20,21^ strong spin splitting,^20^ and magnetic field-mediated tunable Dirac and Weyl states.^23^ These properties, together with the feasibility in obtaining the atomically thin crystals, make this material family a versatile platform for investigating exotic phenomena of relativistic Fermions in nanostructures. In this work, taking advantage of the suppressed bulk contributions in the exfoliated ZrSiSe flakes, we have successfully probed transport of the surface floating band. Unlike the topological nontrivial surface states in many other topological nodal point semimetals, such a floating surface band is topologically trivial^4^ but surprisingly exhibits quantum oscillations with high mobility, which is not generally expected. The robustness of the surface state, as demonstrated both from our transport measurements and density functional theory (DFT) calculations, pave a way for surface-related device applications in quantum computing and spintronics.

**Figure 1:**
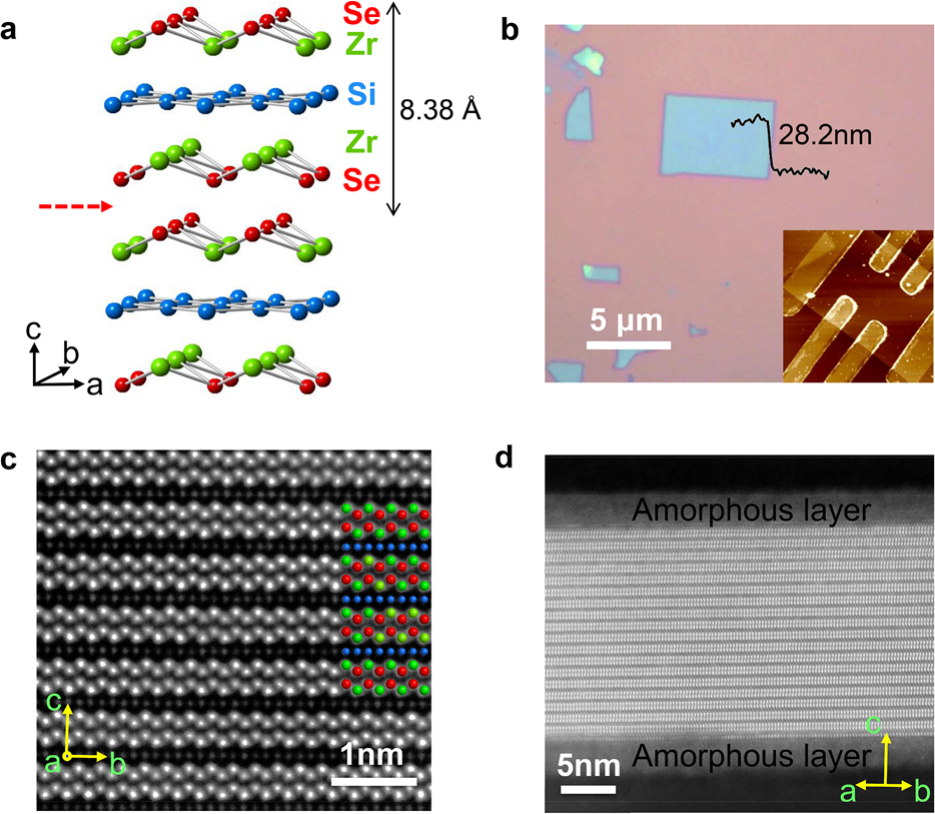
ZrSiSe crystal structure and microscopy characterizations. (a) Crystal structure of ZrSiSe, showing the Se–Zr–Si–Zr-Se slabs and the cleavage plane (red arrow). (b) Optical microscope image of a 28.2 nm ZrSiSe nanoflake on Si/SiO_2_ wafer obtained through micromechanical exfoliation. Inset, atomic force microscope image of a Hall bar device. (c, d) Atomic resolution annular dark-field (ADF) aberration-corrected scanning transmission electron microscopy (STEM) images of (c) the bulk along the [100] zone and (d) exfoliated ZrSiSe flakes along the [110] zone. Inset in c, the [100] zone (cross-section) image matches well with the crystal structure.

Figure 1a shows the crystal structure of ZrSiSe, which can be viewed as the stacking of Se–Zr–Si–Zr–Se slabs. The weak interslab binding strength allows for the mechanical exfoliation of ZrSiSe to atomically thin layers,^21^ as demonstrated in Figure 1b. The atomic resolution scanning transmission electron microscope (STEM) images of the as-exfoliated flakes reveal good crystallinity for the inner parts (Figure 1c) with shallow amorphous oxidation layers (∼5 nm) on the top and bottom surface (Figure 1d and Figure S1). The stacking of Zr, Si, and Se atoms precisely matches the expected lattice structure of ZrSiSe (Figure 1c, inset).

ZrSiSe devices (Figure 1b, inset) are fabricated through the standard electron beam lithography. With the magnetic field applied perpendicular to the sample surface (i.e., along the *c*-axis), we observed clear Shubnikov-de Haas (SdH) oscillations in magnetoresistance (MR) for all ZrSiSe thin flakes with various thickness at low temperatures (see Figure S3). Surprisingly, the oscillatory components of the longitudinal resistivity Δ*ρ*_*xx*_, obtained by subtracting background, exhibit different signatures between very thick and thin flakes. In Figure 2a, we present the Δ*ρ*_*xx*_ for typical thick (176 nm) and thin (28.2 nm) samples. For the thicker sample (176 nm), the oscillation pattern contains only a single frequency of *F*_B_ = 210 T as revealed by the fast Fourier transform (FFT) analysis (Figure 2a, inset), which is consistent with the observation in the single crystal bulks.^21^ In contrast, the oscillation pattern of the thinner sample (28.2 nm) deviates from the “bulk-like” behavior (Figure 2a) with an additional frequency occurring around *F*_S_ = 445 T (Figure 2a, inset). Although *F*_S_ appears to be close to 2 × *F*_B_, it should not be regarded as a harmonic frequency of *F*_B_ because of their distinct angular dependencies, as will be shown later. Such a surprising, additional frequency component is reproducible for all thin flakes below 60 nm (see Figures S4 and S5). It is worth noting that multiple frequencies in quantum oscillations in bulk *WHM* crystals have been discovered,^20,21,30,32−34^ but our observation of the emergence of a new frequency upon reducing thickness is unique.

**Figure 2:**
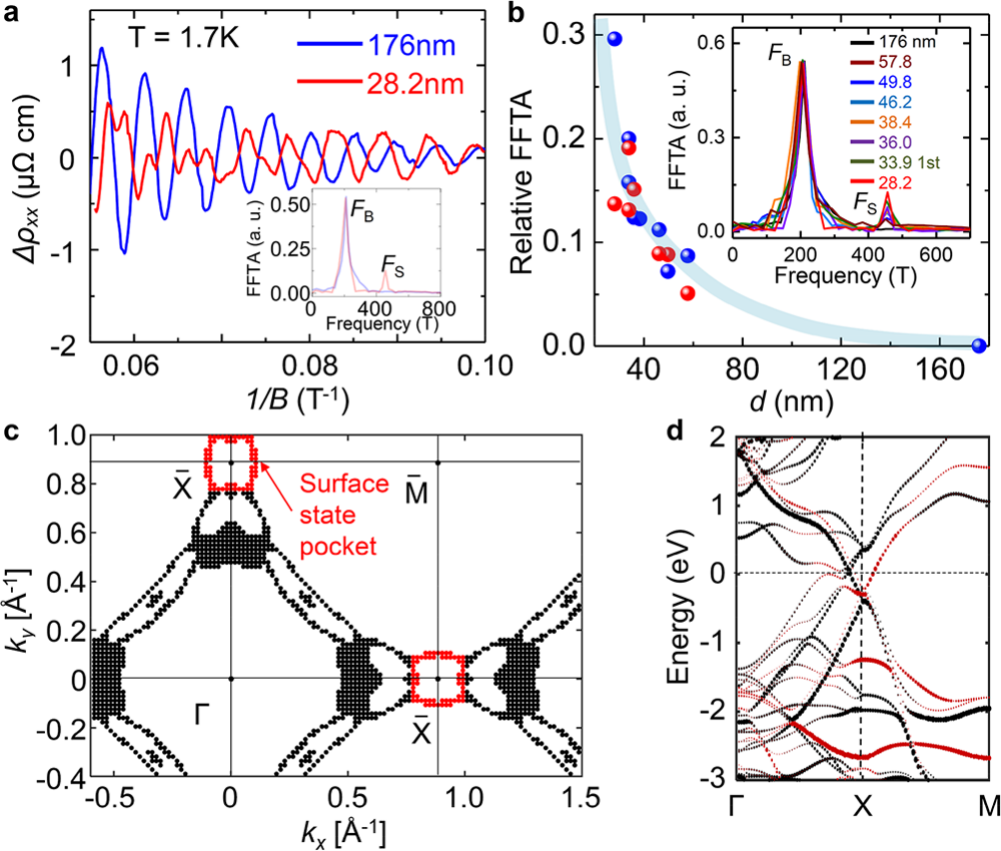
Thickness-dependent SdH oscillations. (a) Oscillatory components Δ*ρ*_*xx*_ of thick (176 nm) and thin (28.2 nm) ZrSiSe samples with magnetic field normal to the sample surface. Inset, the fast Fourier transform of the corresponding oscillation patterns. The FFT for the 28.2 nm sample is normalized to the 176 nm sample according to the *F*_B_ for clarity. The additional frequency of *F*_S_ = 445 T appears for the thin sample. (b) Thickness dependence of the relative FFT amplitude (FFTA) between *F*_S_ and *F*_B_ bands. The blue and red solid dots are based on the longitudinal and transverse resisitivity analysis, respectively. Inset, corresponding FFT spectra for different thicknesses, normalized to the 176 nm sample according to the *F*_B_ peak and only the first 33.9 nm sample is included for clarity, other FFT analysis can be found in the Supporting Information. (c) Calculated Fermi surface cross-section at *k*_*z*_ = 0 of a three-layer ZrSiSe. The surface Fermi pocket is labeled in red. (d) Calculated energy band dispersion of a three-layer slab ZrSiSe near X. The red color denotes the contribution from the surface state.

In principle, a quantum oscillation pattern with a specific frequency corresponds to an extremal Fermi surface cross-section. Therefore, the additional frequency in thin samples indicates that an additional electronic band starts to play a substantial role in transport only in the samples with reduced thickness. The modification of band structure due to 2D quantum confinement is widely observed in 2D materials by reaching the monolayer limit. However, it is unlikely that quantum confinement takes effect at a thickness of ∼60 nm, where the *F*_S_ component already becomes visible (Figure 2b). Instead, this unusual frequency is most likely a manifestation of a new surface state owing to a few characteristics. First of all, the signal weight of the *F*_S_ band in the transport measurement grows with the decreasing sample thickness. As shown in Figure 2b, the relative ratio between the FFT peak amplitudes of *F*_S_ and *F*_B_ increases significantly when the flake thickness is reduced, indicating the increased weight of the *F*_S_ component in thinner samples. This result agrees well with the surface origin of the *F*_S_ band and is a natural consequence of the enhanced surface-to-bulk ratio with reducing the thickness, which has also been observed in Cd_3_As_2_.^13^

In addition, the 2D character of the *F*_S_ band is in line with a surface state. As shown in Figure 3a, for a typical sample with a moderate thickness of about 36 nm, the SdH oscillation weakens when the magnetic field is rotated away from the perpendicular direction (θ = 0°), which is consistent with observations in bulk ZrSiSe^21^ and other *WHM* compounds.^30,33,35^ However, as shown in Figure 3b, the angular dependences of *F*_B_ and *F*_S_ obtained from FFT are entirely different: *F*_S_ varies significantly with θ, which is distinct from the very weak angular-dependence of *F*_B_, and indicates it is not a second-order harmonic of *F*_B_. Such angular dependences for *F*_B_ and *F*_S_ are highly reproducible with various sample thicknesses (Figure S5). To better illustrate the angular dependences of both frequencies in various samples, we have summarized the data in the polar plot shown in Figure 3c. *F*_B_ (blue) appears to be nearly θ-independent up to θ = 45°, consistent with the previous studies on bulk samples.^21^ In contrast, *F*_S_ at various θ obtained from different samples are well-aligned to a vertical line (red dashed lines) in the polar plot, i.e., showing a 1/cos θ dependence. Such a 1/cos θ dependence implies 2D nature for the *F*_S_ band, expected for surface state.^13^

**Figure 3:**
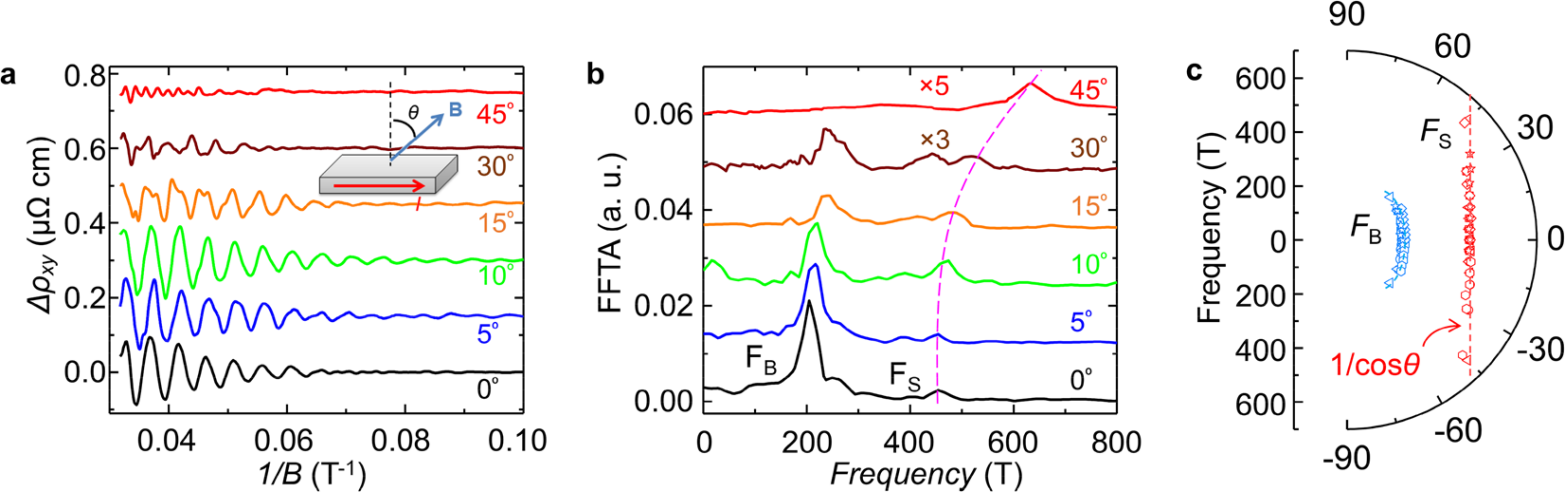
Angular-dependent SdH oscillations. (a) Angular-dependent oscillatory components Δρ_*xy*_ of the 36 nm ZrSiSe device at *T* = 0.6 K. Inset, the measurement setup. (b) Fast Fourier transform of the SdH oscillation pattern shown in panel a, with a field range 1/*B* from 0.0318 to 0.1 T^–1^. Note: the 30 and 45° spectra have been multiplied by 3 and 5 times, respectively. (c) Polar plot of the angular-dependence of bulk frequency *F*_B_ (blue) and surface frequency *F*_S_ (red) from all measured devices (see Figures S4 and S5 for oscillation patterns and their FFTs). The dashed straight line (red) indicates the 1/cos θ dependence for *F*_S_.

Furthermore, the agreement of the oscillation frequency with our DFT calculations and previous ARPES observations^36,37^ provides further support for the surface origin of *F*_S_. According to the Onsager relation (*F* = *ℏA*/2πe), the observed *F*_S_ frequency at θ = 0° corresponds to a Fermi surface cross-section area *A* of 4.25 nm^–2^, which matches well with the area of the ellipse-like surface-derived electron pocket around the Brillouin zone X point estimated in our DFT calculations (∼4.32 nm^–2^, Figure 2c), as well as that probed in ARPES experiment (∼4.58 nm^–2^, estimated from ref (36).). Given no other Fermi pocket with comparable size can be found either in our DFT calculations or ARPES reports,^36,37^ the *F*_S_ frequency most likely reflects such an electron pocket of a surface-related state inferred in *WHM* compounds.^4^

Now, we discuss the mechanism of forming such a surface band. Generally, a surface state is expected to be formed as a result of the termination of the bulk potential or surface defects/adsorbates in conventional materials. This possibility can be easily excluded because quantum oscillations, which rely on the formation of complete cyclotron orbits and high mobility (i.e., sharp Landau levels), are generally not expected for “dirty” materials. Given defects or adsorbates are strong scattering centers, quantum oscillation from a surface state is often easily destroyed in conventional materials. However, in ZrSiSe, *F*_S_ and its angular dependence in thin flakes are prominent and highly reproducible, even with significant amorphous surface layers observed by STEM (Figure 1d). Such observations are clearly inconsistent with extrinsic origins such as surface degradation, unintentional doping, and strain effect.

In addition, in a typical *nodal-point* topological semimetal with isolated bulk Dirac or Weyl points, the bulk-boundary correspondence principle results in a gapless mode at the edge when the symmetry group protecting the topology of bulk bands is unbroken on the edge.^3^ However, this possibility can also be excluded. ZrSiSe and related *WHM* compounds exhibit the coexistence of nodal-line and nodal-point Dirac states protected by different symmetries,^16,17,26^ but neither of them should lead to a topological surface state. In *WHM* compounds, topological surface states originated from band inversion-induced Weyl-like states^38^ and other gapless Dirac state^39^ have been reported. However, a topological surface state arising from the *gapped* nodal-line band has not been revealed in either first-principles calculations^17^ or ARPES experiments.^16,18,36^ Similarly, a topological surface state relevant to the nodal-point Dirac state arising from the bulk-boundary correspondence principle is not expected, as the corresponding nonsymmorphic symmetry is not preserved at the (001) plane of the crystal.^3^

After ruling out the possibility of surface chemistry and bulk-boundary correspondence, we argue that this robust *F*_S_ surface band revealed in our quantum oscillation experiments represents the recently proposed novel floating surface states derived from the surface symmetry reduction in nonsymmorphic semimetals.^4^ Topp et al. showed that the ZrSiS bulk symmetry with nonsymmorphic space group *P*4/*nmm* is reduced to the symmorphic wallpaper group *P*4*mm* at the natural cleavage (001) surface. Such nonsymmorphic symmetry reduction significantly deforms the orbital, which lifts the degeneracy of the bulk bands at Brillouin zone X point and consequently causes an unpinned surface band floating on top of the bulk band.^4^ Such a proposed floating surface state is quantitatively consistent with the ARPES observations of the Fermi pocket with the 2D character at X.^4,16^ The isostructural compound ZrSiSe studied in this work also exhibits electron pocket at X point with similar surface states, as revealed by our DFT calculations (see Figure 2c, d and Supplementary Note 1).

The properties of the *F*_S_ band provide further support for this argument. The surface floating band is formed by lifting the degeneracy of the bulk band and is thus topological trivial,^4^ which can be revealed by the Berry phase analysis. We have separated the *F*_B_ and *F*_S_ oscillation components and extracted the Berry phase for both bands using the Landau fan diagram (see Methods). As shown in Figure 4a, for ZrSiSe flakes with a range of thickness, the linear fits of the Landau indices *n* yield intercepts *n*_0_ around 0 and −0.5 for *F*_S_ and *F*_B_ bands, respectively. Berry phase ϕ_B_ can be derived via 2π(*n*_0_+δ), where δ = ± 1/8 for the 3D band (e.g., the bulk *F*_B_ band) and 0 for the 2D band (e.g., the surface *F*_S_ band). As summarized in Figure 4b, the Berry phase is trivial (ϕ_B_^surface^ ≈ 0) for the surface *F*_S_ band in each sample, in sharp contrast with that of the bulk *F*_B_ band which exhibits an average Berry phase of ϕ_B_^Bulk^ ≈ −0.68π ± 1/4 π. This result is further verified through directly fitting the oscillation pattern using the multiband Lifshitz-Kosevich model (see the Supporting Information), which confirms the distinct topology of the bulk and the surface floating bands in ZrSiSe.

**Figure 4:**
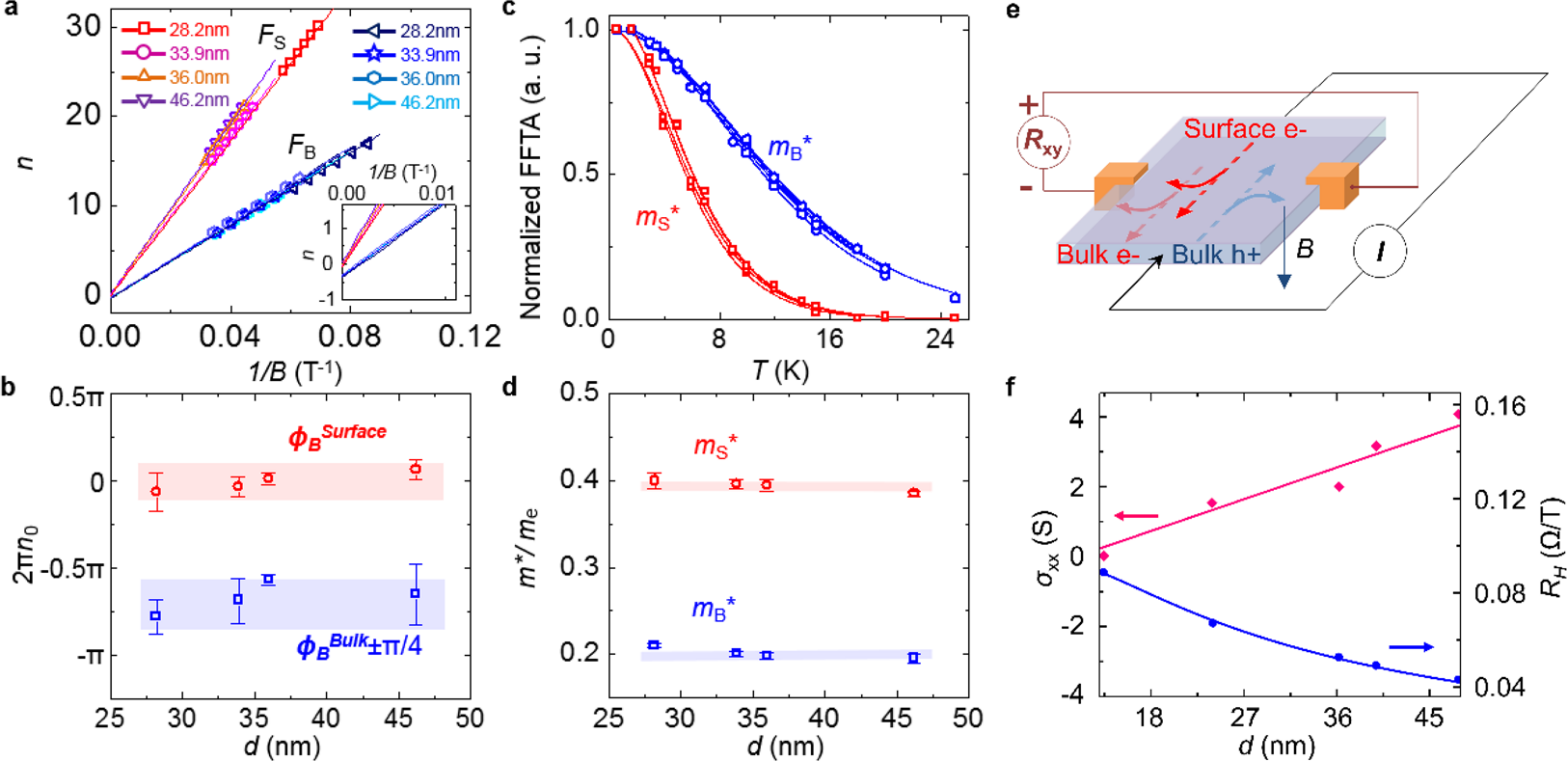
Property comparison between the bulk and surface bands. (a) Landau Level (LL) fan diagram for the bulk *F*_B_ and surface *F*_S_ states of four ZrSiSe nanodevices with thicknesses of 28.2, 33.9, 36, and 46.2 nm. The solid lines represent linear fits of the Landau indices, which intercept around 0 for the surface band and −0.34 for the bulk band. Inset, zoom-in view showing different intercepts for surface and bulks. (b) Berry phases derived from the LL fan diagram shown in (a) for samples with different thicknesses. (c) Temperature dependence of the FFT amplitude for bulk and surface bands for the same four samples in panel a. The solid lines indicate the fits to the thermal damping term of the LK-model. (d) Effective masses for bulk and surface states derived from the fitting shown in panel c. (e) Schematic drawing of the multichannel contributions to the Hall effect. Here, bulk e-, bulk h+, and surface e- denote contributions from bulk electron, bulk hole, and surface electron, respectively. (f) Thickness-dependent longitudinal conductivity and Hall coefficient. The solid lines show the fits to the three-channel model (see the Supporting Information).

Furthermore, the effective cyclotron mass of the *F*_S_ band also agrees with the scenario of the surface floating band. The formation of the floating band at the surface of our material can be modeled by breaking the nonsymmorphic glide plane symmetry and introducing a large mass for S and Zr orbitals,^4^ so such surface state is expected to be more massive, which is indeed observed in ZrSiSe. We have extracted effective masses for both bulk *F*_B_ and surface *F*_S_ bands from the temperature dependence of FFT amplitude for ZrSiSe samples with various thicknesses (see Methods) (Figure 4c). As summarized in Figure 4d, the effective cyclotron mass for the surface floating band *m*_S_* is around 0.39*m*_0_ (*m*_0_ denotes free electron mass) for all analyzed samples, which is around twice as large as that of the bulk band (*m*_B_* ∼ 0.19*m*_0_).

The above discussions have established that the additional *F*_S_ component observed in the quantum oscillation of ZrSiSe nanoflakes originates from the surface floating band. The observation of quantum oscillations caused by such topological trivial surface state is unusual because the lack of a protection mechanism is generally expected to lead to a vulnerable surface state with low mobility that is not favorable to quantum oscillations. Despite of the apparent surface degradations (Figure 1d and Figure S1), the LK-fitting (see the Supporting Information) has revealed high quantum mobility of 1.20 × 10^3^ cm^2^ V^–1^ s^–1^ at 1.7 K for the topologically trivial surface *F*_S_ band, which is comparable with the topologically protected bulk band (1.74 × 10^3^ cm^2^ V^–1^ s^–1^). The high quantum mobility for the surface band is consistent with the transport mobility of 1.84 × 10^3^ cm^2^ V^–1^ s^–1^ estimated from the multichannel model of Hall effect (Figure 4e, f) (see the Supporting Information). This result implies minimized surface scattering of charge carriers caused by surface deformation or disorders. Indeed, it is consistent with our STEM observations in Figure 1d, which shows an atomically sharp interface between the oxidized amorphous layer and the inner crystalline layer. The formation of such amorphous layer also explains why the ARPES observations varies strongly with the sample preparation methods. Because ARPES is an extremely surface sensitive technique, so that any oxidation or degradation of the topmost layer may significantly affect the spectra, as has been observed in ref.^40^ However, the surface floating band originates from the symmetry breaking at the surface so that it can exists even in the presence of surface oxidization. As discussed in Supplementary Note 2, the surface band is still preserved even by replacing all the Se atoms in the topmost surface with [OH] or [O_4_]. Therefore, in our samples, such surface state can occur at the interface between the inner crystalline layer and the outer amorphous layer where the bulk symmetry is broken, and manifest in quantum oscillations in thin flakes. The robustness of the surface band itself is unexpected and may benefit from a sort of protection mechanism that deserves further investigations. One possible interpretation could be the connection with the bulk topological band: given the surface floating band for nonsymmorphic ZrSiSe is caused by lifting degeneracy of the bulk band at the surface, it could be robust when the corresponding bulk band is topologically protected. In ZrSiSe, the surface floating band is related to the bulk Dirac band protected by the nonsymmorphic symmetry. Therefore, an “indirectly” protected surface bands with trivial topology could appear in ZrSiSe, which represents a novel protection mechanism in crystalline solid with similar nonsymmorphic symmetry.

In summary, we have systematically studied quantum oscillations of exfoliated ZrSiSe nanoflakes and successfully detected a new 2D, trivial surface state, which can be attributed to the surface floating state caused by symmetry reduction at the surface. Our results also suggest such a surface is trivial but robust and likely protected via a new mechanism. Our findings provide a new arena for the study of exotic surface states in topological quantum materials, which is an important step toward practical application in modern electronics and surface-related devices such as quantum computing and spintronics.

## Methods

### Sample Preparation

The ZrSiSe single crystal was synthesized by using a chemical vapor transport (CVT) method. The stoichiometric mixture of Zr, Si, and Se powder was sealed in a quartz tube with iodine being used as a transport agent (2 mg/cm^3^). Platelike single crystals with metallic luster can be obtained via the vapor transport growth with a temperature gradient from 950 to 850 °C. The composition and phase of the single crystals were examined by energy-dispersive X-ray spectroscopy and X-ray diffraction, respectively. The thin flakes of ZrSiSe were obtained through a micromechanical exfoliation. The thickness of thin flakes was precisely determined by an atomic force microscope. The ZrSiSe devices with the standard four-terminal resistivity or six-terminal Hall bar geometry were fabricated by using the standard electron beam lithography, followed by the deposition of 5 nm Ti/50 nm Au as contacts via electron beam evaporation. Ohmic contacts of devices were achieved by current annealing before the transport measurement (see the Supporting Information).

### Scanning Transmission Electron Microscopy

Atomic-resolution annular dark field STEM images of the flakes were recorded with a JEOL ARM200F over collection angles 45–180 mrad. High signal-to-noise images were formed by averaging multiple, rapidly acquired frames to remove scan distortions.

### Magnetotransport Measurements

Before the high field experiments, the ZrSiSe devices were tested by an in house 9T-PPMS. The high field magnetotransport measurements were performed at National High Magnetic Field Laboratory (NHMFL) in Tallahassee by using an 18T superconducting magnet and a 31T resistive magnet. The AC current used for all devices was between 20 and 50 μA supplied by Keithley 6221 AC and DC Current Source. The longitudinal/transverse voltages were measured using lock-in amplifiers with the frequencies triggered by the AC currents. The noise ratio was reduced by twisted pairs between two voltage cables and two current cables, respectively.

### Landau Level Fan Diagrams

To examine the Berry phase ϕ_B_ accumulated along cyclotron orbits for bulk and surface bands, we performed Landau Level (LL) fan diagram analysis using the longitudinal conductivity *σ*_*xx*_, which was derived via *σ*_*xx*_*= ρ*_*xx*_*/(ρ*_*xx*_^2^+ρ_*xy*_^2^) where *ρ*_*xx*_ and *ρ*_*xy*_ are longitudinal and transverse resistivity, respectively, as shown in Supplementary Figure S6. Quantum oscillations arising from bulk and surface bands are separated by FFT filters to build the LL fan diagram for each band. We assigned the integer LL index to the oscillation maximum of *σ*_*xx*_ according to the previous quantum oscillation study on this family of materials,^20^ and extracted Berry phase for each band from the intercept of the linear fit of the LL fan diagram. Detailed analysis is provided in the Supporting Information. Although there are debates on the assignment of the integer LL indices, we intend to emphasize an observed Berry phase difference between bulk and surface band, which does not depend on the way of assigning integer LL indices and implies distinct topology of the bulk and the surface floating bands in ZrSiSe.

### Effective Mass

The effective masses of bulk and surface bands for various samples were obtained from the temperature dependence of the quantum oscillations (1.7 to 20 K), as shown in Figure S10, by fitting the FFT peak intensity to the thermal damping term of the LK-formula.^41^

## Supporting Information Available

Materials include cross-sectional STEM and corresponded element mapping analysis, current annealing process, details of all measured samples and corresponding data analysis, in-plane conductivity and hall conductivity analysis, Berry phase analysis, transport mobility analysis, temperature-dependent FFT spectra, DFT calculations of the ZrSiSe band structure and surface state, and robustness discussions of the ZrSiSe surface state (PDF)
