# Supplementary material for: Quantum Transport of the 2D Surface State in a Nonsymmorphic Semimetal
Source: Nano Lett. 2021 Apr 9;21(11):4887–93. doi: 10.1021/acs.nanolett.0c04946 (PMC8193637; doi:10.1021/acs.nanolett.0c04946)
Supplement: Supplementary file 1 — nl0c04946_si_001.pdf [file nl0c04946_si_001.pdf]

Supplementary Information

Quantum transport of the 2D surface state in a nonsymmorphic semimetal

Xue Liu[1,2]*, Chunlei Yue[2], Sergey V. Erohin[3,4], Yanglin Zhu[2], Abin Joshy[2], Jinyu Liu[2], Ana M Sanchez[5], David Graf[6], Pavel B. Sorokin[3,4], Zhiqiang Mao[7,2], Jin Hu[8]*, Jiang Wei[2]*

[1]Institutes of Physical Science and Information Technology, Anhui University, Hefei 230601, China

[2]Department of Physics and Engineering Physics, Tulane University, New Orleans, Louisiana 70118, USA

[3]National University of Science and Technology "MISIS", Leninsky prospect 4, Moscow 119049, Russian Federation

[4]Moscow Institute of Physics and Technology (State University), 9 Institutskiy per., Dolgoprudny, Moscow Region, 141701, Russian Federation

[5]Department of Physics, University of Warwick, Coventry, CV4 7AL, United Kingdom

[6]National High Magnetic Field Lab, Tallahassee, Florida 32310, USA

[7]Department of Physics, Pennsylvania State University, University Park, Pennsylvania 16802, USA

[8]Department of Physics, Institute for Nanoscience and Engineering, University of Arkansas, Fayetteville, Arkansas 72701, USA

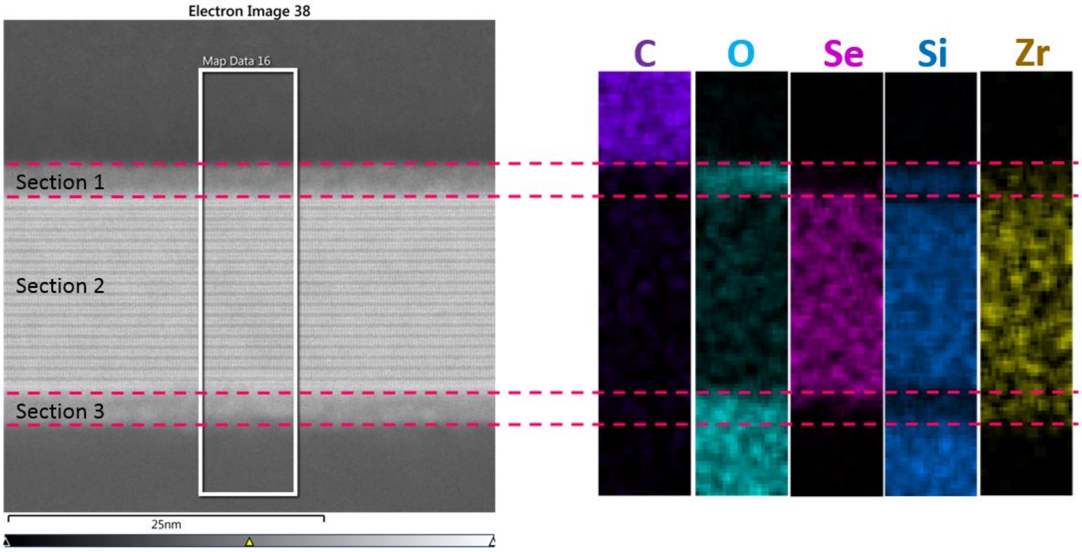

Supplementary Figure S1. Atomic resolution annular dark-field (ADF) cross-sectional scanning transmission electron microscopy (STEM) image (left image) of as exfoliated ZrSiSe thin flake and corresponded element mapping (right image) through Energy Dispersion X-ray Spectroscopy (EDX) analysis. Note: the ZrSiSe flakes are exfoliated in ambient environment onto Si wafer, a layer of carbon has been evaporated on the top surface to improve the conductivity for imaging. Section 1 and section 3 indicate clear amorphous oxidation layers on the top and bottom surface of ZrSiSe crystal, as confirmed by the EDX mapping.

Current annealing process

The fabricated ZrSiSe devices were conducted current annealing between every two electrodes of the Hall bar device geometry, which has been used for cleaning or improving contacts of graphene devices[1,2]. By using a parameter analyzer, a DC voltage was applied and swept step by step from 0V up to 2.5V with the current recorded. A typical annealing process has been shown by Supplementary Fig. S2, before annealing the I-V sweeps show nonlinear behavior with high resistance ~0.4 MΩ indicating a large contact resistance caused by the oxidation layer between the metal and ZrSiSe single crystals; when the voltage sweeps up around 2.4V, the current suddenly jumps to 1 mA which is the preset current limitation. Then, after stabilizing for a few sweeps, the I-V sweep becomes linear, and the two-

probe resistance decreases down to ~1 KΩ. By processing current annealing, the oxidation layer under the electrodes has been broken down to achieve nearly ohmic contact between metal and ZrSiSe crystals.

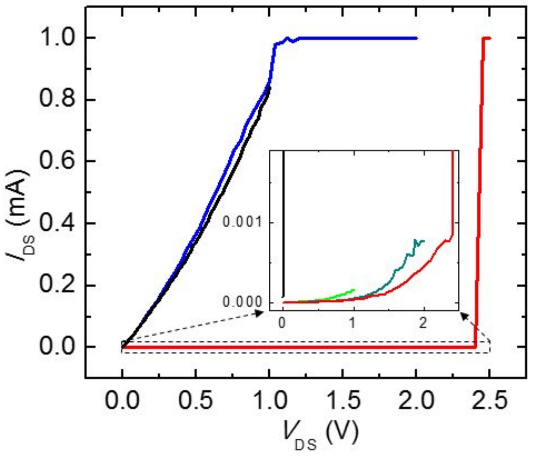

Supplementary Figure S2. I_{DS}-V_{DS} sweeps during current annealing process for a typical ZrSiSe device. Inset, zoom-in view of the non-linear I-V sweeps before successful annealing. Note: the red curve exhibits a current "jump" from 0.001mA to 1mA at 1V bias (the current limitation preset in the parameter analyzer), indicating a breakdown of the oxidation layer between the metal and ZrSiSe crystal. The black curve shows the final linear dependence after successful annealing.

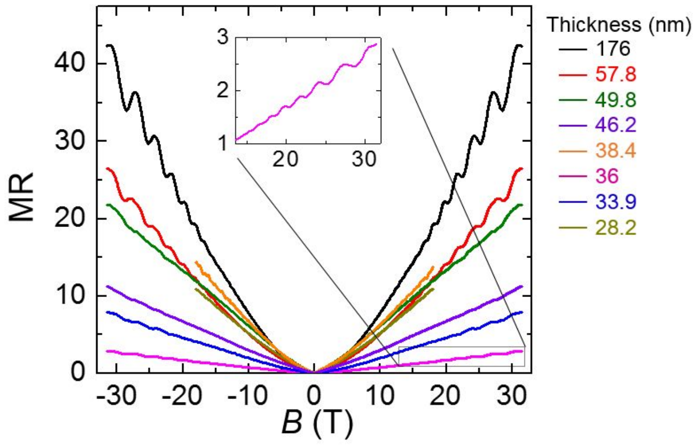

Supplementary Figure S3. Magnetoresistance (MR) $[\rho(B)\text{-}\ \rho(B\text{=}0)]/\rho(B\text{=}0)$ at T = 1.7 K of exfoliated ZrSiSe samples with different thicknesses. The magnetic field up to 31T is applied perpendicular to the surface (*i.e.* along the *c*-axis) of different samples, except for 38.4nm and 28.2nm samples. The SdH oscillations are clearly seen, as shown in the inset.

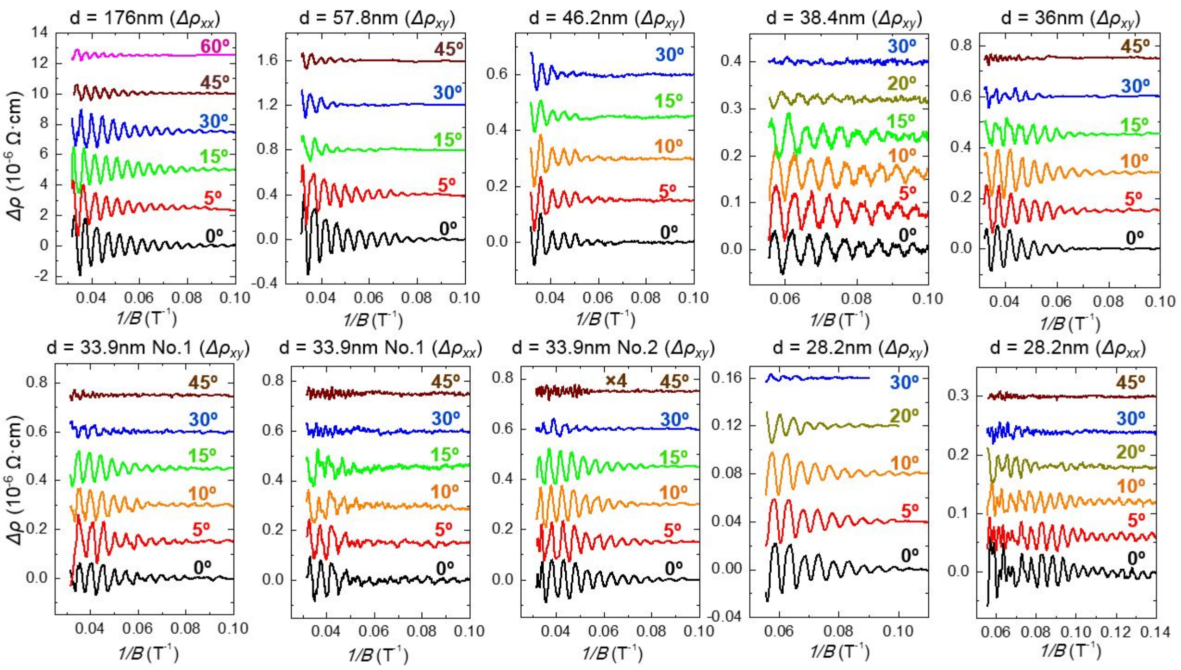

Supplementary Figure S4. Shubnikov-de Haas (SdH) oscillations at T = 1.7 K under different magnetic field orientations for samples with various thicknesses. Data for different angles have been shifted for clarity. The oscillatory components are extracted from either longitudinal (ρ_{xx}) or transverse (ρ_{xy}) resistivity.

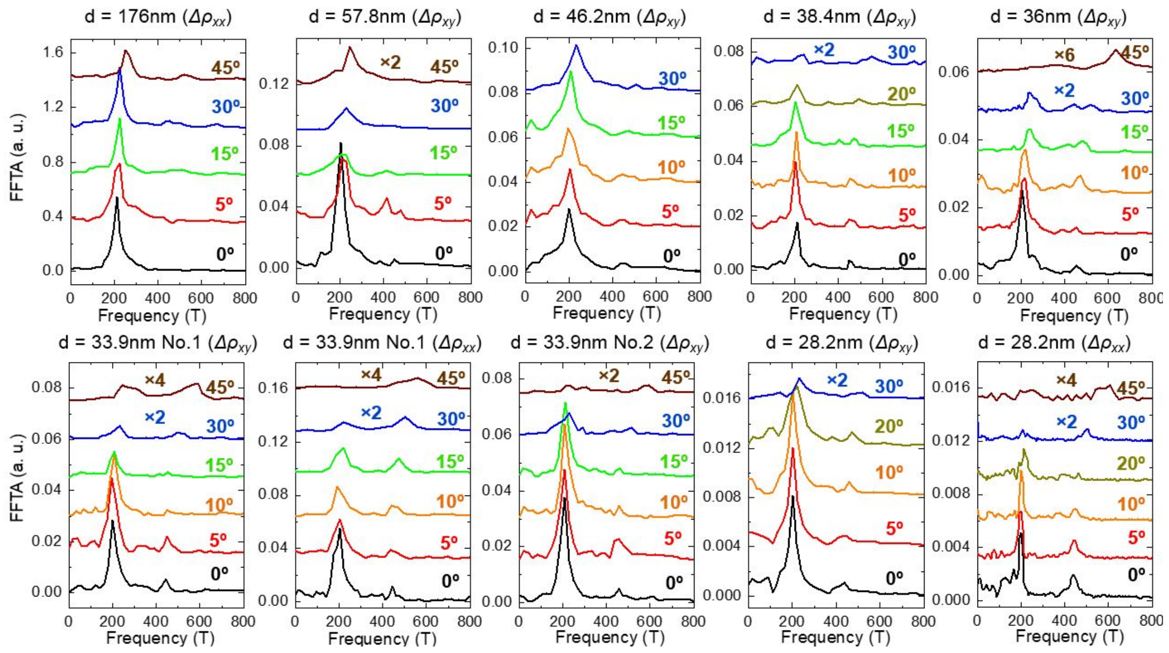

Supplementary Figure S5. Fast Fourier transform amplitude spectrums under different magnetic field orientations for oscillations in Supplementary Fig. S2. Data for different angles have been shifted for clarity. The F_S peak is not observable for the bulk-like 176 nm sample, but become more pronounced with decreasing the sample thickness.

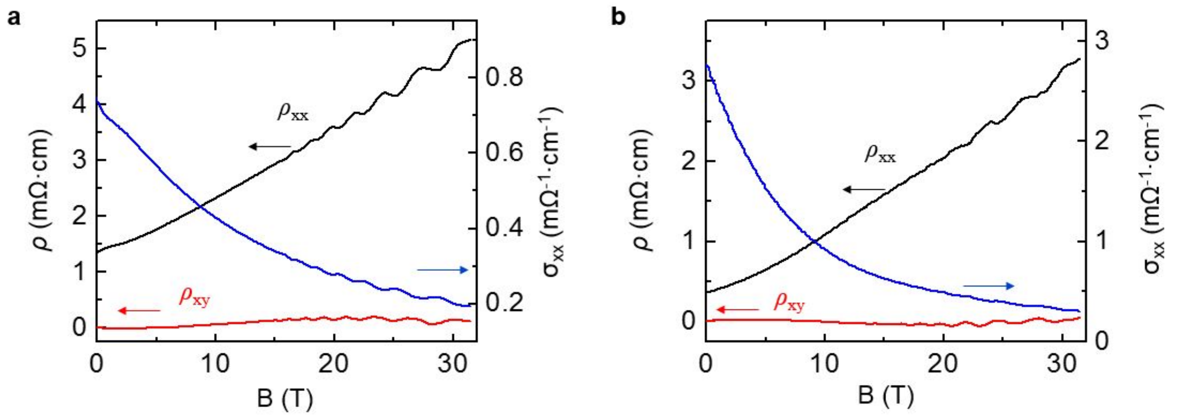

Supplementary Figure S6. The longitudinal (black), transverse (red), and conductivities (blue) for (**a**) 36 nm and (**b**) 33.9 nm devices. The conductivity is obtained from the tensor conversion: $\sigma_{xx} = \rho_{xx}/(\rho_{xx}^2 + \rho_{xy}^2)$.

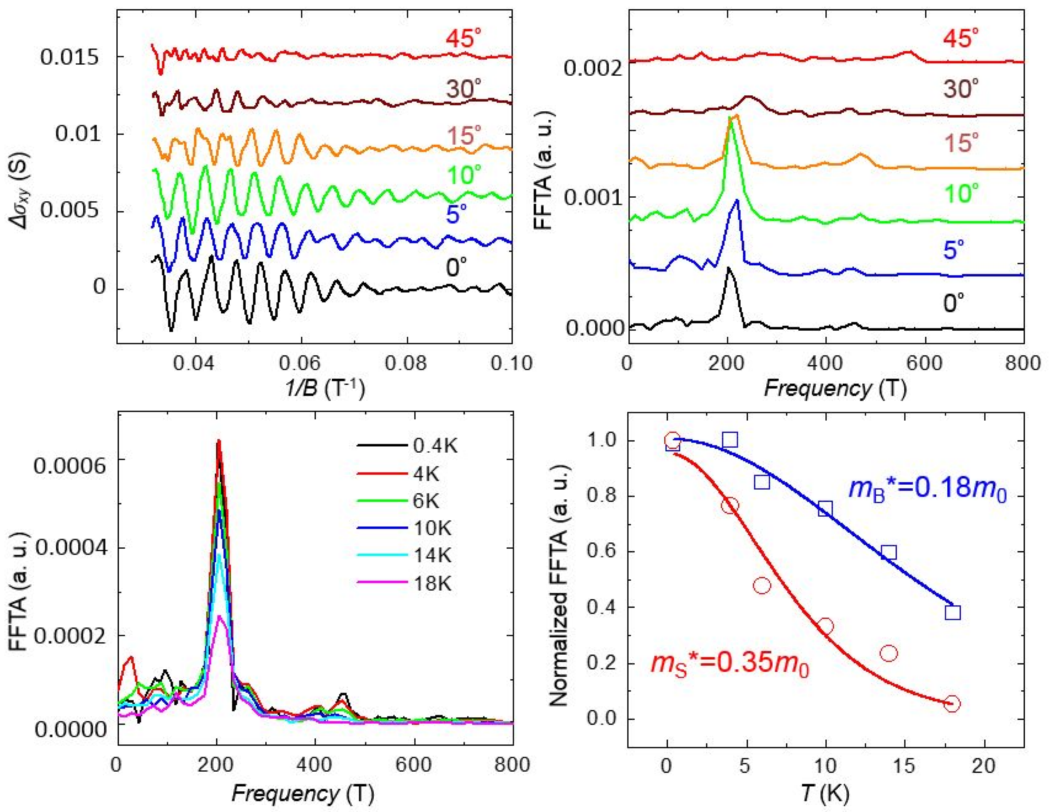

Supplementary Figure S7. Hall conductivity analysis for the 36nm sample, including angular dependent SdH oscillations and FFT spectra, temperature FFT spectra, and effective mass estimations. Note, the results show great consistency with that from longitudinal resistivity and transverse resistivity analysis.

Berry phase analysis

Berry phase of the bulk and surface band was extracted by Landau level (LL) fan diagrams and the Lifshitz-Kosevich fit. LL fan diagrams were built from quantum oscillation in conductivity, derived via $\sigma_{xx} = \rho_{xx}/(\rho_{xx}^2 + \rho_{xy}^2)$. As shown in Fig. S8 below, oscillations arising from bulk and surface bands were separated by FFT filters to establish the LL fan diagram for each band. Integer LL indices were assigned to the oscillation maxima of σ_{xx} according to the previous quantum oscillation study[3]. Berry phase for each band was extracted from the intercept of the linear fit of the LL fan diagram (see main text). The slope of each linear fit yields the oscillation frequency, which only differs from the frequency obtained from the FFT by 1-2%. Such consistency indicates the reliability of the obtained intercept and the derived Berry phase.

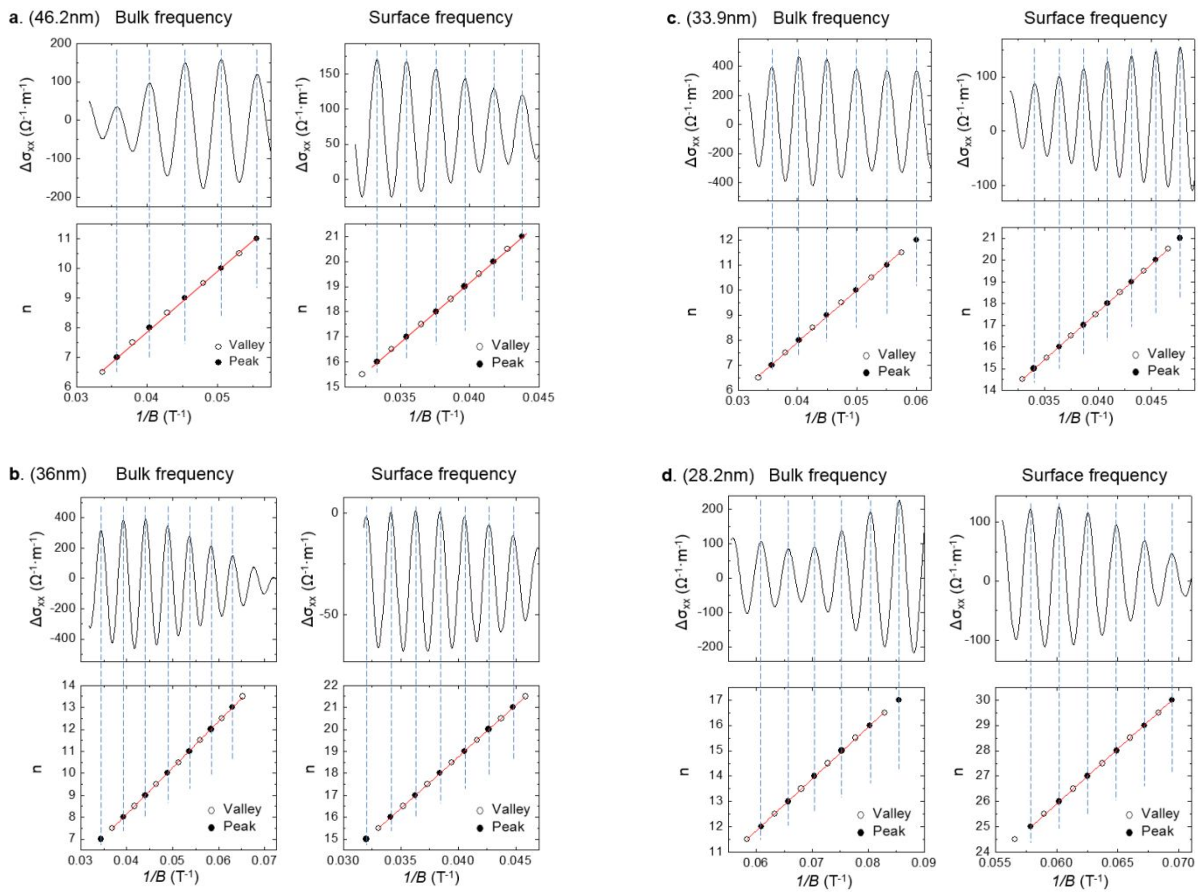

Supplementary Figure S8. Landau Level index fan diagram for (**a**) 46.2nm, (**b**) 36nm, (**c**) 33.9nm, and (**d**) 28.2nm samples.

In addition to the LL index fan diagram, we also performed the direct Lifshitz-Kosevich (LK) fitting to extract the Berry phase. In our ZrSiSe system, the SdH oscillations are treated as the linear superposition of bulk and surface frequency (F_B, F_S) oscillations. Each frequency component can be described as[4]:

$$\Delta\rho_{xx} = A\frac{5}{2}\left(\frac{B}{2F}\right)^{\frac{1}{2}} e^{-\lambda_D}\frac{\lambda}{\sinh\lambda}\cos\left[2\pi\left(\frac{F}{B}+\gamma-\delta\right)\right]$$

The thermal damping factor $\lambda = 2\pi^2 k_B T m^*/(\hbar eB)$, $\lambda_D = 2\pi^2 k_B T_D m^*/(\hbar eB)$, where m^* is the effective mass, T_D is Dingle temperature, F is the frequency, γ is defined as $\gamma = 0.5 - \phi_B/2\pi$, and δ is the phase shift, which is $\pm 1/8$ for 3D and 0 for 2D. Typical fittings for 36 nm and 46.2 nm samples are shown in Supplementary Fig. S9. The extracted Dingle temperature for bulk and surface states are 6.49 K and 4.57 K, respectively, from which the quantum relaxation time $\tau_q = \hbar/(2\pi k_B T_D)$ and quantum mobility $\mu_q = e\tau_q/m^*$ can be extracted. Moreover, the fitting also yields Berry phases of -1.09π (δ = -1/8) or -0.59π (δ_{3D} = 1/8) for the bulk and 0.1π (δ_{2D} = 0) for the surface. These values are in good agreement with those derived from the LL fan diagram.

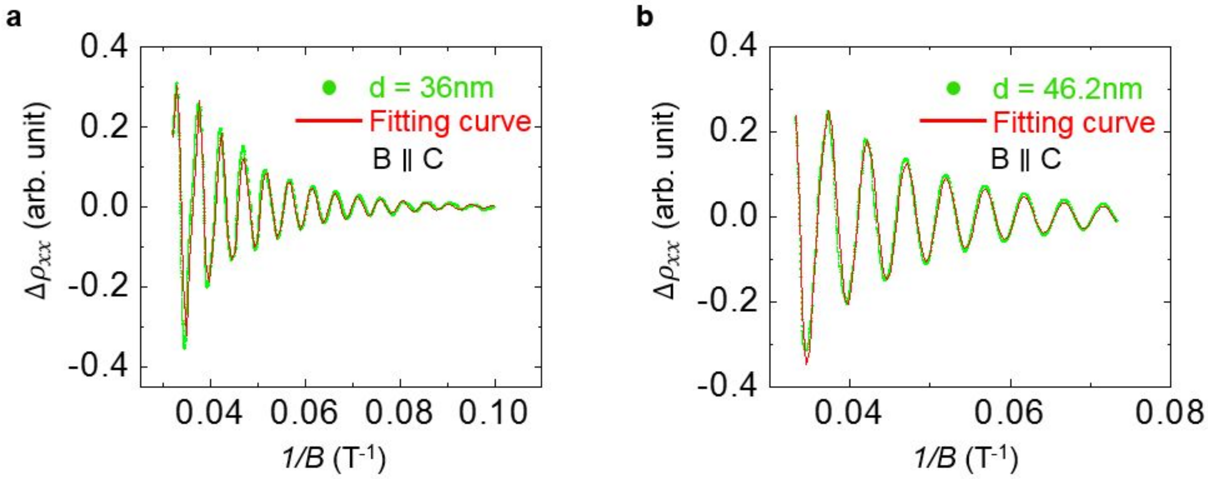

Supplementary Figure S9. Lifshitz-Kosevich fittings for (**a**) 36nm and (**b**) 46.2nm samples.

Transport mobility from Hall effect

As illustrated in Fig. 4e, the Hall signal is contributed from bulk electron, bulk hole, and surface electron (as confirmed by ARPES measurements[5]). Then, the conductivity and hall coefficient can be described by a three-channel model[6]:

$$\sigma_{xx} = en_p\mu_p - en_n\mu_n - en_t\mu_t,$$

$$R_H = \frac{n_p{\mu_p}^2 - n_n{\mu_n}^2 - n_t{\mu_t}^2}{e[n_p\mu_p + n_n\mu_n + n_t\mu_t]^2}$$

with $n_n = n_e \times d$, $n_p = n_h \times d$. Here, n_e, n_h are the hole and electron carrier density of bulk state, respectively, and d is the sample thickness. n_t is the surface state carrier density. μ_n, μ_p, μ_t are bulk hole, bulk electron, and surface electron mobilities, respectively. By fitting the thickness dependent σ_{xx} and R_H (as plotted in Fig. 4f), we can extract μ_t=1.84×10^3 cm^{2}V^{-1}s^{-1} for the surface band and μ_p=1.39×10^4 cm^{2}V^{-1}s^{-1}, μ_n=1.03×10^4 cm^{2}V^{-1}s^{-1} for bulk band. These derived quantum and transport mobilities of the surface electrons are comparable with that of the topological protected bulk electrons in many other topological semimetals[7]. The fitted surface carrier density is n_t=5.1×10^{13}cm^{-2}, and bulk carrier density n_e=0.67×10^{20}cm^{-3} and n_h=0.32×10^{20}cm^{-3}. From the SdH frequency F_S=445T, we can calculate the corresponding 2D surface carrier density by using $n_{2D} = \frac{Fg'e}{h}$, where h is the Planck constant, g' is the degeneracy factor, and e is the electron charge. Taking g' = 4 because of a spin degeneracy of 2 and both top and bottom surface contribute to the carrier density, we can obtain a 2D surface carrier density of 4.3 × 10^{13}cm^{-2}, which agrees with the fitted n_t.

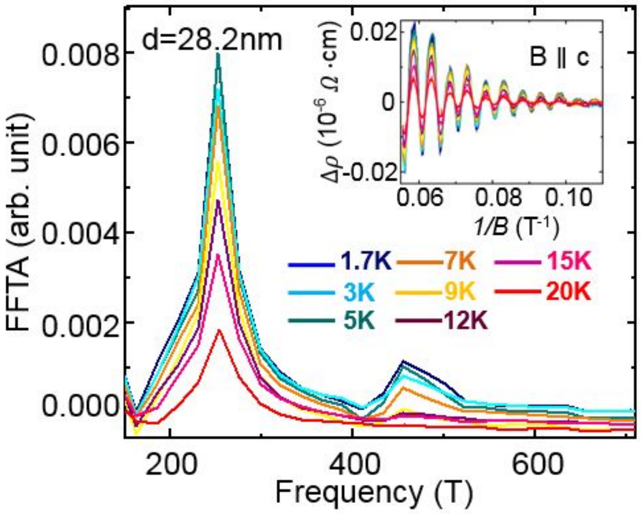

Supplementary Figure S10. Temperature dependent FFT spectra for the 28.2nm sample. Inset, the oscillation patterns at different temperatures.

Supplementary Note 1. DFT calculations of the ZrSiSe band structure and surface state.

We also performed DFT calculations of the electronic band structure of the ZrSiSe crystal surface. All calculations were carried out within the Perdew-Burke-Ernzerhof exchange correlation energy functional (PBE)[8] implemented in Vienna ab initio simulation package (VASP)[9-11]. The plane-wave cutoff energy of 400 eV was used. To ensure sufficient calculation accuracy, the Brillouin zone sampling was made by the Monkhorst-Pack scheme[12] with 8×8×4 grid for the 3D case and 8×8×1 grid for the slab structure. A vacuum size of 15 Å was set along the c axis, which is perpendicular to the slab planes, to simulate 2D structures inside the VASP periodic cell. The unfolding of supercell band structure has been performed using the BandUP code[13, 14].

To understand the experimental observation of a floating band, we performed slab model calculation for a thickness of six unit cells along the c lattice parameter, see Supplementary Figure S11a. The bulk bands arising from inner layers are clearly seen in the band structure. The consequent increasing of the vacuum space between SeZr-Si-ZrSe layers leads to the shift of the electron band at

X-M with final fall down by ~1 eV (Figure 1-right image) with forming of two electron pockets centered at X in the slab. This arises from the Zr 4d orbitals in the slab. In bulk ZrSiSe, these Zr 4d orbitals are bonded with the Se p orbitals along the (001) direction (see wave function distribution in the Supplementary Fig. S11b left inset) whereas in the slab Zr d orbitals are connected only with Si p orbitals (see wave function distribution in the Supplementary Fig. S11b right inset). Indeed, this surface state has been observed in its isostructural compound ZrSiS which has been attributed to an unusual floating surface band[15].

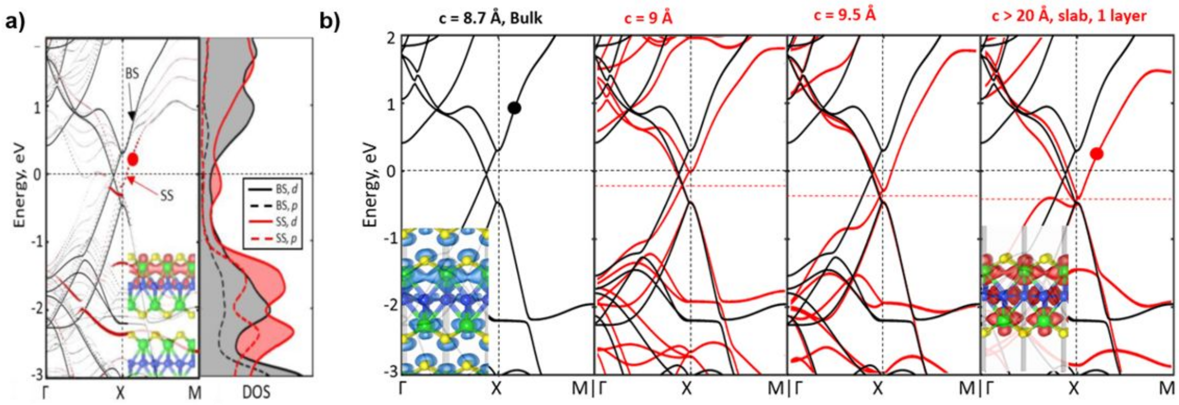

Supplementary Figure S11. (a) Electronic band structure and corresponded density of state calculations for a slab model consisting of six ZrSiSe unit cells. **(b)** The evolution of the bulk band structure (marked by black) into slab band structure (marked by red) with consequent increasing of the vacuum space between SeZr-Si-ZrSe layers. Insets show the structure and the distribution of the wave function corresponding to the marked band and k-point. Se, Zr and Si atoms are marked by yellow, green, and blue colors.

Supplementary Note 2. Robustness discussions of the ZrSiSe surface state.

One can tune the floating band by changing the chemical environment of Zr through surface decoration/coverage or by interface formation. In Supplementary Fig. S12a we present the termination of the surfaces, which destroys the floating band or lifts it higher Fermi level. However, the termination of the dangling bonds leads to the restoration of the floating band (Supplementary Fig. S12b). A general

observation is that the surface contained a lot of dangling bonds (Supplementary Fig. S12a), i.e., in case of unrealistic Si termination of ZrSiSe crystal, produces surface states dramatically different from that in bulk or the slab. Besides, the second ZrSe (near-surface) layer contributes only to bulk bands. The possible tuning of the floating band is originated from its trivial character, which has been discussed in the previous report[15].

On the other hand, it is highly likely that the interface between the oxidation layer and ZrSiSe observed in the experiment weakly affects the floating band. We simulated an oxide layer as the zirconium orthosilicate in which O surrounds the Zr and Si atoms. We simulated the interface between the double-layered slab of ZrSiSe supercell (2×2×1 unit cells) and the $ZrSiO_4$ layer (Supplementary Fig. S12c). The DFT calculations clearly show that the $ZrSiO_4$ only weakly interacts with the ZrSe surface due to its high chemical stability, which leads to the preservation of the floating band crossing the Fermi level. Therefore, the $ZrSiO_4$ bands do not appear near the floating band states due to their sizeable insulating bandgap. One can propose that the amorphous oxidation layer does not affect the band structure of ZrSiSe and protect the floating band from any other chemical modifications.

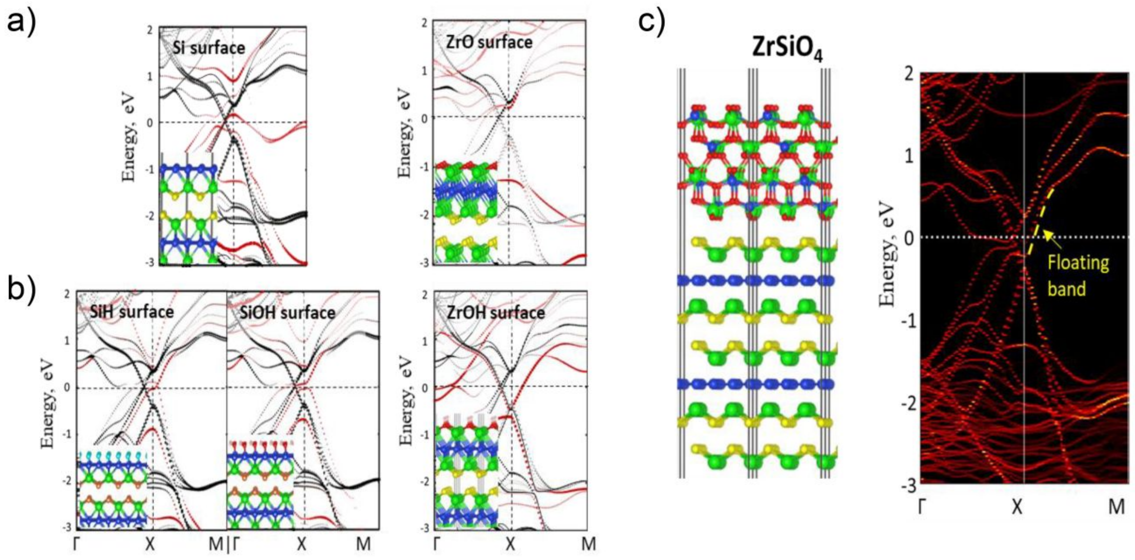

Supplementary Figure S12. Robustness discussions of the ZrSiSe surface state. (**a**) DFT calculated electronic band structures of different terminated surfaces, including Si surface and ZrO surface. (**b**) Restoration of the floating surface states by dangling bonds termination. (**c**) Band structure of a double-layer ZrSiSe slab with a top oxidation layer $ZrSiO_4$.

Reference

1. Moser, J., Barreiro, A., & Bachtold, A. Current-induced cleaning of graphene. *Appl. Phys. Lett.* **2007**, 91, 163513.
2. Ramamoorthy, H., & Somphonsane, R. In-situ current annealing of graphene-metal contacts. *J. Phys.: Conf. Ser.* **2018**, 1144, 012186.
3. Hu, J., *et. al.* Nearly massless Dirac fermions and strong Zeeman splitting in the nodal-line semimetal ZrSiS probed by de Haas-van Alphen quantum oscillations. *Phys. Rev. B* **2017**, 96, 045127.
4. Shoenberg, D. (Cambridge Univ. Press, **1984**).
5. Hosen, M. M., *et. al.* Tunability of the topological nodal-line semimetal phase in ZrSiX-type materials (X=S, Se, Te). *Phys. Rev. B* **2017**, 95, 161101.
6. Backes, D., *et. al.* Disentangling surface and bulk transport in topological-insulator p-n junctions. *Phys. Rev. B* **2017**, 96, 125125.
7. Ando, Y. Topological Insulator Materials. *J. Phys. Soc. Jpn.* **2013**, 82, 102001.
8. Perdew, J. P., Burke, K., & Ernzerhof, M. Generalized gradient approximation made simple. *Phys. Rev. Lett.* **1997**, 77, 3865.
9. Kresse, G. & Furthmuller, J. Efficient iterative schemes for ab initio total-energy calculations using a plane-wave basis set. *Phys. Rev. B* **1996**, 54, 11169.
10. Kresse, G. & Furthmuller, J. Efficiency of ab-initio total energy calculations for metals and semiconductors using a plane-wave basis set. *Computational Materials Science* **1996**, 6, 15-50.
11. Kresse, G. & Hafner, J. Ab initio molecular-dynamics simulation of the liquid-metal-amorphous-semiconductor transition in germanium. *Phys. Rev. B* **1994**, 49, 14251.
12. Monkhorst, H. J. & Pack, J. D. Special points for Brillouin-zone integrations. *Phys. Rev. B* **1976**, 13, 5188.
13. Medeiros, P.V.C., Stafstrom, S. & Bjork, J. Effects of extrinsic and intrinsic perturbations on the electronic structure of graphene: Retaining an effective primitive cell band structure by band unfolding. *Phys. Rev. B* **2014**, 89, 041407.
14. Medeiros, P.V.C., Tsirkin, S.S., Stafstrom, S. & Bjork, J. Unfolding spinor wave functions and expectation values of general operators: Introducing the unfolding-density operator. *Phys. Rev. B* **2015**, 91, 041116.
15. Topp, A., *et. al.* Surface floating 2D bands in layered nonsymmorphic semimetals: ZrSiS and related compounds. *Phys. Rev. X* **2017**, 7, 041073.
